# Mass Spectrometric Fingerprinting to Detect Fraud and Herbal Adulteration in Plant Food Supplements

**DOI:** 10.3390/molecules30143001

**Published:** 2025-07-17

**Authors:** Surbhi Ranjan, Tanika Van Mulders, Koen De Cremer, Erwin Adams, Eric Deconinck

**Affiliations:** 1 Section of Medicines and Health Products, Sciensano, J. Wytsmanstraat 14, B-1050 Brussels, Belgium; surbhi.ranjan@sciensano.be; 2 Department of Pharmaceutical & Pharmacological Sciences, Pharmaceutical Analysis, KU Leuven, Herestraat 49, O&N2, PB 923, B-3000 Leuven, Belgium; erwin.adams@kuleuven.be; 3 Section of Platform Chromatography and Mass Spectrometry, J. Wytsmanstraat 14, B-1050 Brussels, Belgium

**Keywords:** illegal plant food supplements, chemometrics, weight loss supplements, mass spectrometry, fingerprinting

## Abstract

Mass spectrometric (MS) fingerprinting coupled with chemometrics for the detection of plants in plant mixtures is sparsely researched. This paper aims to check its value for herbal adulteration concerning plants with slimming as an indication. Moreover, it is among the first to exploit the full three-dimensional dataset (i.e., time × intensity × mass) obtained with liquid chromatography hyphenated with MS for herbal fingerprinting purposes. The MS parameters were optimized to achieve highly specific fingerprints. Trituration’s (total 55), blanks (total 11) and reference plants were injected in the MS system to generate the dataset. The dataset was complex and humongous, necessitating the application of compression techniques. After compression, Partial Least Squares-Discriminant Analysis (PLS-DA) was performed to generate models validated for accuracy using cross-validation and an external test set. Confusion matrices were constructed to provide insight into the modeling predictions. A complimentary evaluation between data obtained using a previously developed Diode Array Detection (DAD) method and the MS data was performed by data fusion techniques and newly generated models. The fused dataset models were comparable to MS models. For ease of application, MS modeling was deemed to be superior. The future market studies would adopt MS modeling as the preferred choice. A proof of concept was carried out on 10 real-life samples obtained from illegal sources. The results indicated the need for stronger monitoring of (illegal) plant food supplements entering the market, especially via the internet.

## 1. Introduction

The popularity of plant food supplements has risen immensely over the past decade, particularly for slimming or weight loss supplements. Obesity occurs due to a lack of exercise, a sedentary lifestyle, high-calorie intake, or medical conditions (for example, diabetes) [1]. Supplements have been conventionally used for the treatment of obesity, even though very little is known about their safety and efficacy. This, coupled with the obsession with a ‘slim’ body standard aligning with societal norms, has led to these products having a high demand in the market. Consequently, their immense use has led weight–loss supplements to be one of the highest adulterated classes [2,3,4,5].

The increase in acceptability and consumption of such products can be highly attributed to the notion that everything ‘natural’ is ‘safe’, therefore altering consumer perception. This paradigm shift can be reasoned through various facilitating factors such as the awareness around side-effects of allopathic medicines, the ability to self–medicate, the ease of procuring these products without prescription, the influence of various media outlets (e.g., radio, and print media) and the increasing market for such products on the internet [6,7,8,9]. The evolution of internet marketing allowed a whole new channel for these products from all over the world to enter the market. However, as the demand for these products increased, it rendered them vulnerable to malpractices by the various source vendors. These malpractices can be economically motivated or accidental [10,11,12,13] and can broadly be classified as intentional (e.g., adulteration or fraud) or unintentional (e.g., confounding). Further, it branches into chemical adulteration and herbal adulteration or fraud. While chemical adulteration is the addition of chemical substances to the product, herbal adulteration refers to the substitution of the part/whole (medicinal) plant that reduces the quality and efficacy of the plant product (fraud) or the addition of forbidden toxic plants or regulated plants without disclosing them on the packaging (adulteration). In both categories, the consumer is unaware of the content of the products they are consuming, therefore further risking consumer trust and health and necessitating the need to exercise legislative actions/interference to control such products.

The European (Union) market for plant food supplements is regulated by the European directive 2002/46/EC [14]. It permits the sale of plant food supplements following market authorization by the national authorities of the member nations that validate the concerned safety standard requirements. Concerning Belgium, the Royal Decree of 1997 provides a list of plants/plant parts allowed to be used or marketed in food supplements. This decree consists of three lists: list 1 comprises forbidden plants/plant parts for use, list 2 consists of edible mushrooms, and list 3 dictates the name of the plants/plant parts allowed for usage and can enter the Belgian market after mandatory authorization from the responsible organization [15].

The identification of crude plant materials is generally carried out through macroscopic and microscopic evaluations. However, this approach is not useful in the case of plant mixtures containing multiple plants that are pulverized and pressed in the form of capsules, tablets, etc. The World Health Organization (WHO) and Chinese Pharmacopoeia recognize chromatographic fingerprinting as a method for the identification and quality evaluation of plants [16,17,18]. Fingerprinting can be defined as the unique chemical profile of the plant/plant material that can be determined through chromatographic, spectroscopic or electrophoretic techniques [19], where chromatographic fingerprinting is by far the most popular. However, chromatographic fingerprinting might pose a problem in mixtures with a more complex plant matrix, but this can be solved using chemometric tools for better identification and interpretation of complex data [17,18,20].

Chemometrics is a tool to extract information from chemical data using mathematics and statistics [21]. It has been used in combination with chromatographic fingerprinting to identify and authenticate plant materials with quality markers [22,23,24,25]. However, it is in its infancy when applied to complex herbal matrices [25]. The literature available is even less for MS practices concerning plant mixtures with chemometrics. The research group previously developed a multidimensional ultra-high performance liquid chromatography (UHPLC)-DAD method coupled with chemometrics for the identification of regulated plants in illegal plant food supplements [20], and as a continuation, this paper will explore a liquid chromatography (LC)-MS method for the same.

The paper explores a novel approach, where LC-MS is not used for the identification of some quality parameters or markers, but where it is used to generate a fingerprint of the sample, for the identification of targeted plants with chemometric modeling in order to tackle the problem of potential herbal adulteration or fraud with plant food supplements. The initial analysis was carried out using the previously developed UHPLC-DAD method [20], followed by an optimization step. The raw data generated by MS was three-dimensional (i.e., time points × intensity × mass), providing more specificity to the chromatograms compared to ultraviolet (UV) chromatograms. Triturations were prepared using botanical supplements (that did not claim any weight-loss indication) and the reference plants mixed in five varying concentrations, which were then injected to create the dataset. The dataset was subjected to alignment with correlation optimized warping (COW), pretreatment, and compression (to reduce the size of the dataset) before chemometric analysis. Partial least squares-discriminant analysis (PLS-DA), as a supervised modeling technique, was applied for the classification of the regulated plants in plant food supplements. The selection of four regulated plants was based on the popularity of the plant, the experience of the laboratory and the Rapid Alert System for Food and Feed (RASFF). The plant, *Aristolochia fangchi*, fell into list 1 of the Royal Decree of 1997 (forbidden for sale in Belgium), whereas *Ilex paraguariensis*, *Hoodia gordonii* and *Garcinia cambogia* fell into list 3 of the Royal Decree, thus meaning they are allowed to be commercialized after authorization by the national authorities. A proof of concept was also carried out with 10 selected illegal samples to assess the MS method and explore the possibility of herbal adulteration in these samples. In addition, a complementary analysis comparing the two techniques, i.e., DAD method [20] and MS method, along with a fused data set (DAD and MS), was performed to assess the best method for future market surveillance studies.

## 2. Results

### 2.1. MS Development

MS measurements were carried out using a quadrupole time of flight (Q-ToF) mass analyzer following a UHPLC system. The preliminary gradient and choice of the chromatographic method for recording the mass fingerprints were based on the previously used UHPLC–DAD method [20]. The method was further optimized to obtain selective and specific MS chromatograms with maximum information, meaning a maximum number of separated peaks. The MS tune parameters were optimized, including the capillary voltage (ranging from 2.5 kV to 3.5 kV), source temperature (100–150 °C), sampling cone (100–150 °C), and the desolvation temperature, which was held constant at 500 °C as per the instrument and could not be altered. Gas flows were adjusted within specified ranges: cone flow (50–100 L/h), desolvation gas (500–900 L/h), and nebulizer pressure (4–7 bars). The final conditions are listed in Table 1 and Table 2 The optimization process began with default settings as instructed by the manufacturer (Waters) for the chosen flow rate, which were then further refined. A full scan was run in positive mode with a mass range between 50 and 2000 m/z. The accepted error in mass tolerance was 5 ppm. An effort to find one common method for all the plants was made. A compromise method could be devised for three plants, *Aristolochia fangchi*, *Ilex paraguariensis* and *Hoodia gordonii*. In contrast, *Garcinia cambogia* produced a more characteristic profile with a different gradient based on the comparison of the Total Ion Chromatograms (TIC) obtained.

**Figure 1:**
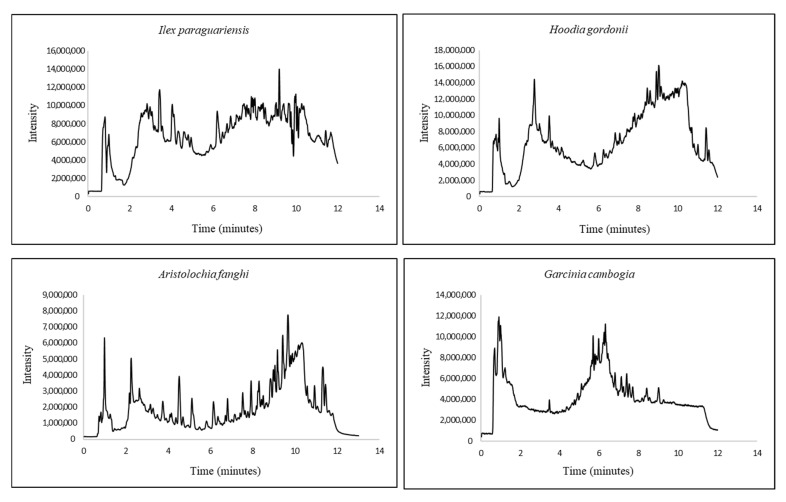
MS chromatograms for the four regulated plants.

The mode of operation for both methods was high resolution in positive mode and common MS tune parameters. It was revealed that the default tune parameters provided the best results. The total ion chromatograms for all four plants obtained with their final methods can be found in Figure 1.

### 2.2. Chemometrics—MS

The constantly developing technological advancements of analytical instruments have led to higher scientific capabilities. Consequently, the data produced by these instruments has also grown in complexity, rendering the approach to treating raw MS data not so straightforward.

The raw data obtained from the system were treated in Progenesis software and then exported to Excel. In Excel, the data were arranged and further imported into MATLAB (version 2020), and a dataset consisting of timepoint × intensity × mass data was created.

The dataset was then subjected to COW to reduce the inter- and intra-day differences in the measurements. Preprocessing techniques such as Savitsky–Golay derivatization were explored and selected as part of the approach if this resulted in better predictive models.

The MS measurements produced a humongous amount of data, therefore necessitating the use of compression techniques. After exploring different possibilities, PLS compression was carried out, and classification models were constructed using PLS-DA. Compression helped in reducing the data size and provided ease of treatment. PCA compression was also evaluated, but modeling did not yield better results than PLS compression (see Supplementary Materials, Tables S1–S4). As a result, only PLS results are presented.

The dataset was split into a training and a test set using the duplex algorithm. Out of 67 samples (55 triturations + 11 blanks/negatives + 1 reference), 25% were added to the test set (17 samples), and the rest formed the training set (50 samples). Binary modeling was carried out using PLS-DA, and classes were assigned as ‘1’ for positive and ‘2’ for negative for the targeted plant, respectively. Cross-validation using ‘10-fold cross-validation’ was performed for models with a complexity up to 30 PLS factors. The lowest number of PLS factors with a maximum correct classification rate (ccr%) in cross-validation was selected, and modeling was performed. An external test set validation was carried out to validate the selected models.

The Variable Importance in Projection (VIP) scores were evaluated, and significant signals (at specific time points) could be detected (see Supplementary Materials Figures S1–S4). However, it was difficult to interpret the plot as we used the information obtained from the whole fingerprint for further modeling. The difficulty in evaluating the plots also arose due to the complexity of these plant matrices.

The results for PLS-DA modeling and the confusion matrices are represented in Table 3 and Table 4 for the four plants.

The best results for *Aristolochia fangchi* after preprocessing using the first derivative were obtained using three PLS factors. The cross-validation results depicted no misclassification in the training set, making up a percentage of 100%. The model showed a calibration ccr% of 100%. It was validated using an external test set, and a score of 100% was obtained with all samples predicted correctly.

For *Ilex paraguariensis*, the data set showed a cross-validation ccr% of 100% with all correct predictions. The modeling results also gave a ccr% of 100%. The models were tested, and a ccr% of 94% for the external test set prediction was found, with one trituration misclassified as a negative. On further evaluation, this sample was found to be of a lower concentration, amounting to 1/15.

For *Hoodia gordonii*, the dataset was also derivatised, and then PLS-DA was applied. The ccr% for cross-validation, modeling, and external validation was revealed to be 100% for both the training and test sets.

In the data set for *Garcinia cambogia* after the application of PLS-DA, a cross-validation ccr% of 98% was determined, with one misclassification identified as a false positive. The modeling score was assessed to be 94%. Furthermore, the model was validated, and a ccr% of 94% was obtained for the external test set, also with one misclassification identified as 1/20 in the data set. This sample was identified as a false negative and can be explained by the presence of a lower concentration of reference plant material in the prepared trituration.

Even though it was an effort to tackle the MS data, the approach provided good results that were validated using external test set validation for all of the plants.

### 2.3. Complimentarity Evaluation/Data Fusion

After carrying out detection through both techniques, i.e., DAD and MS, an approach to explore the complementary nature of these two techniques was defined. Mid-level fusion and high-level fusion were applied to the DAD data and MS data.

In mid-level fusion, compression by PCA and then treatment with PLS-DA was carried out. The DAD data and MS data are both compressed by PCA separately and then merged into one dataset by mid-level data fusion. On the other hand, in high-level fusion, a compression based on PLS was carried out, followed by PLS-DA modeling.

A comparison was made between these two techniques, and it was illustrated that the high-level fusion technique provided comparatively better results. Furthermore, all three models studied until now, i.e., DAD models [20], MS models and fused models, were compared and are represented in Table 5. The differences, i.e., improvement in modeling, were then studied.

After analyzing the modeling results for the three different approaches, it was revealed that MS models were slightly more accurate than DAD and fused models. This can be observed from Table 5. While comparing the external test set predictions for the four plants, a near-perfect %ccr was observed for all MS models. However, if DAD models are compared with MS models, the results are also quite acceptable. On the other hand, while results for the fused data sets were on par with MS results, developing fused models did not provide any improvement into the modeling results. Moreover, creating fused models requires injections of the samples in UHPLC-DAD and LC-MS systems, subsequently increasing the experimental time and effort. These factors were significant while considering the application of fused models, and MS modeling provided the best model outcomes. Hence, the latter was the choice for future market surveillance studies and thus for the following proof of concept.

### 2.4. Proof of Concept for Real–Life Samples

The above-developed MS modeling technique was applied to 10 real-life samples, which were selected from a collection of samples seized by the Federal Agency for Medicines and Health Products and the Federal Agency for the Safety of the Food Chain (FAMPH and FASFC) and sent to the laboratory for evaluation of chemical adulteration. All samples showed slimming as their indication.

The results of the screened samples are summarized in Table 6. Samples 1, 5, 7, 8 and 10 claimed the presence of *Garcinia cambogia* on the packaging. Through modeling techniques, it was predicted that *Garcinia cambogia* could not be determined in the selected sample set. This result was in contrast with the fact that the presence of *Garcinia cambogia* in slimming supplements is quite well-known.

Samples 3 and 4 claimed the presence of *Ilex paraguariensis,* which was confirmed by the modeling approach. However, it was also detected in samples 1, 2 and 8. This was not surprising and indicated the popularity of this plant in the illegal market.

No sample claimed the presence of *Aristolochia fangchi*. It is a prohibited plant for sale in Belgium, but surprisingly, it was detected in Sample 8. However, as the origin of these samples is unregulated, the presence of the plant might not be surprising. *Hoodia gordonii,* on the other hand, was not claimed on the packaging of the selected sample set and, after modeling, was only detected in Sample 1.

It was evaluated that, although the modeling approaches were commendable, various other factors such as the concentration of plants in a mixture and their source of procurement need to be considered when making a final decision about the presence or absence of a plant. Modeling results are not 100% accurate, and thus, results should always be confirmed by a different technique, as is good practice in forensic analysis.

## 3. Discussions

This research has explored the possibility of combining chromatographic fingerprinting with chemometrics in the domain of plant food supplements comprising plant mixtures using MS.

An MS methodology was established based on previously developed UHPLC-DAD methods [20] with tuning parameters adapted to generate the most informative fingerprints. A common method could be devised for three plants *Aristolochia fangchi*, *Ilex paraguariensis* and *Hoodia gordonii*, whereas *Garcinia cambogia* provided better specificity with another gradient. MS analysis, in theory [26,27,28], is considered more specific than UHPLC-DAD analysis, which was proven by the superiority of the chemometric models obtained in this research.

The MS fingerprints were exported as raw data into Progenesis QI software for data handling, where it was converted into .csv format, suitable for further use. However, it was complicated to operate with this humongous amount of data produced by the machine, necessitating the use of compression techniques to make the data more suitable for analysis in Matlab (which generated an out-of-memory error when the large uncompressed data set was treated). Data compression was carried out by a PLS approach, generously reducing the data size and making it compatible with working in Matlab.

After compression, PLS-DA was applied to the four datasets, respectively, for each plant. The cross-validation, modeling and external test set prediction ccr% were found to be acceptable for all. A small sample set consisting of 10 real-life samples from the illegal market, seized by the agencies (FAMPH and FASFC), was selected as proof of concept. These samples were weight-loss products, which claimed the presence of some of the regulated plants on the packaging. It was interesting to evaluate the presence of *Aristolochia fangchi,* which was detected in one sample, as it is a forbidden plant in Belgium and should not be present in the market. However, the source of these samples arising from the illegal market is very dubious and thus could provide a reason for this occurrence. On the other hand, *Ilex paraguariensis* has a very widespread occurrence in the market as one of the most common occurring products in slimming supplements. This occurrence was coherent with the MS results, where it could be detected in 5 of the 10 samples. For *Hoodia gordonii,* no sample claimed its presence on their packaging, and it could only be detected in one sample. On the other hand, a surprising result was obtained for *Garcinia cambogia*, which is a well-known component in weight loss supplements. It was claimed in five of the samples, but it could not be detected in any of them. An explanation for this result could be that, as these samples are plant mixtures, the presence of *Garcinia cambogia* could be in sparse quantities while the other plants are present in higher concentrations, thus overpowering the fingerprint of *Garcinia cambogia* in the samples and providing a negative detection result. Another reason could be the similarity in the fingerprints of the plant with another or many plants present in the blend. It should be kept in mind that real-life samples were used as blank matrices to prepare triturations and create models, though it is impossible to cover the wide range of matrices present in the plant food supplement market and incorporate them in the models. The analysis revealed that Samples 1 and 8 each contained two regulated plant species: Sample 1 contained *Ilex paraguariensis* and *Garcinia cambogia*, while Sample 8 contained *Ilex paraguariensis* and *Aristolochia fangchi* that were not claimed on the packaging. This finding demonstrates that multiple adulterants can be present in a single sample. The binary classification models developed in this study were designed to detect each plant species independently, enabling them to successfully identify the presence of multiple regulated plants within the same sample.

Even though MS modeling provided acceptable results, it is well known that no model is 100% accurate. This can be regarded as a drawback in this setting as a plant detected as positive or negative in a sample set has real-life implications for various parties, such as the manufacturer, the regulating bodies, and of course, the consumer, whose health is put at risk due to the unknown compositions. Therefore, this approach proves to be more useful as a pre-screening method, with the highest potential when a high number of samples have to be analyzed. Results should be further confirmed by one or more different techniques. A technique that is being explored in this regard is DNA metabarcoding [29,30] and next-generation sequencing [31], which can differentiate the plants and plant species in a mixture.

Data fusion was carried out to investigate whether combining the two data sets (DAD and MS) would render any improvements in the models. Therefore, a complementary analysis for DAD and MS datasets was performed. Each of these three parameters, i.e., cross-validation, modeling and external test set predictions, was compared for the three datasets. After investigation, it was revealed that the results of the fused dataset were comparable with the MS models, which were slightly better than the DAD models. Therefore, MS modeling was chosen as the preferred approach to be applied in, for example, market surveillance studies. However, in instances where an LC-MS instrument is not accessible to laboratories, DAD could be the method of choice since, as indicated earlier, the results were quite comparable. Moreover, fused models require the injection of samples in both DAD and MS systems, which is more work in the end to obtain comparable results.

## 4. Materials and Methods

### 4.1. Samples and Reagents

Reference plant materials for the four plants, *Aristolochia fangchi*, *Ilex paraguariensis*, *Hoodia gordonii*, and *Garcinia cambogia*, were obtained from the American Herbal Pharmacopoeia (Scotts Valley, CA, USA) together with a certificate of authentication validating the respective plant material.

Reagents such as methanol (MS grade) and water (MS grade) were obtained from Thermo Fischer Scientific (Waltham, MA, USA). Lactose and LC/MS grade formic acid (99.7%) were purchased from Merck (Darmstadt, Germany). The acquity BEH shield column was purchased from Waters corporation (Milford, MA, USA).

Triturations were prepared using samples sent to our laboratory by the FAMPH and FASFC for testing for chemical adulteration and were found negative. A selection was made such that no plants showing the indications discussed in the paper were present in the chosen samples. As proof of concept, 10 samples of botanical mixtures, claiming slimming as an indication and found negative for chemical adulteration, were chosen from those received at the laboratory, also provided by the FAMPH and FASFC.

### 4.2. Sample Preparation

Reference material was powdered by grinding the dried material with a mortar and pestle and then sieved through a sieve with a pore size of 70 µm.

The extractions of the references, triturations and samples were prepared by measuring 10 mg/mL of the sample and diluting it with extraction solvent (methanol and water (50:50, *v*/*v*)). It was then mixed, vortexed and placed in an ultrasonic bath for 40 min. Filtration was carried out using 0.22 µm PTFE and cellulose acetate filters, and then the sample was collected in vials, ready for injection.

### 4.3. Preparation of Triturations

Ten botanical matrices that mainly comprised various blends of plants (+1 lactose) with no indication of slimming potentials were selected for preparing the triturations. The references and the samples were mixed in a ratio of 1/20, 1/15, 1/10, 1/5, and 1/2 with mortar and pestle to homogenize the mixture. A total of 55 triturations were prepared for each of the 4 plants.

### 4.4. LC-HR-MS Instrumentation

MS development was conducted on a Synapt G2 Q-ToF coupled to a UHPLC instrument from Waters (Milford, MA, USA).

The previously developed DAD methods [20] were run on the system to observe and compare the specificity of the chromatograms. The gradient method was optimized to obtain suitable peaks, whereas the MS parameters such as capillary voltage, desolvation gas, and desolvation temperature were varied. The defined parameters for the concerned flow rate provided by Waters made the initial choice of parameters.

The repeatability of the method was evaluated by injecting the reference plants. The analytical sequence included reference plants for each trituration as a quality control step. The three plants that shared a common method were analyzed in duplicate within a single sequence, while for *Garcinia cambogia*, the reference plant, along with the triturations, was analyzed in duplicate using its gradient method. Overall, based on the data from these runs, four distinct models were developed. The final chromatographic methods developed are summarized in Table 1 and Table 2. For *Aristolochia fangchi*, *Ilex paraguariensis* and *Hoodia gordonii*, a compromise method could be selected, though for *Garcinia Cambogia*, an adapted gradient was necessary to ensure specificity.

### 4.5. Dataset Preparation

The recorded MS data were exported into Excel to prepare the datasets. From the obtained chromatograms, a fingerprint region (ranging from 3 to 10 min) was selected for further processing. The dimensions of the 3D matrix comprised time point × intensity × mass data (see Table 7), where, e.g., for *Aristolochia fangchi*, a data cube of 10,680 × 2 × 67 was obtained. Herein, 10,680 represents the time points, 2 represents the 2 columns consisting of information about intensity and mass data, and 67 represents the number of samples. After unfolding, a data set of 67 × 21,360 was obtained. Similar data cubes for the different plants can be studied from Table 7. The classes assigned to the dataset included: class 1 as positive and class 2 as negative for the targeted regulated plant.

Data splitting was carried out to form a calibration (training) set and a validation (test) set to test the suitability of the developed models [32]. Using the Duplex algorithm, the data set comprising 67 samples was split, with 25% of the dataset forming the test set (i.e., 17 samples) and the rest (50 samples) constituting the training set.

### 4.6. COW and Data Pretreatment

COW is one of the most common data alignment techniques used to correct peak shifts in chromatograms. A reference chromatogram is selected, and the unaligned chromatogram is then aligned utilizing the slack and the segment parameters. These slacks and segments are tested in various combinations to achieve the best alignment [32,33]. The same was performed on the dataset as the first step to avoid any inter- and intraday changes.

Pretreatment of data is usually carried out by filtering or creating a ‘clean’ data set. Various techniques, such as autoscaling, Savitsky—Golay derivatization, standard normal variant, and a combination of them as well, could be used for this purpose. These different pre-treatment techniques were explored here in order to find the best approach for modeling the presence of the targeted plants.

### 4.7. Partial Least Squares-Discriminant Analysis (PLS-DA)

PLS-DA is a supervised chemometric technique used for discriminatory and classification purposes. It can be regarded as a highly developed form of principal component analysis (PCA). It performs dimension reduction while taking the response variable, classes in our study, into account. PLS-DA is vulnerable to over-fitting, thus necessitating cross-validation [34] for the selection of the optimal complexity of the model. In this analysis, 10-fold cross-validation was performed, generating models consisting of 1 up to 30 PLS factors, where the lowest number of factors with the highest correct classification rate (ccr%) was selected for modeling. To check the accuracy of the models, an external test set prediction was performed and validated for all the selected models of the four plants. The misclassifications occurring during cross-validation and test set predictions were studied to gather information and trends from the dataset.

### 4.8. Data Fusion Techniques

The term data fusion can be defined as a technique used to combine data from various sources. The idea behind the technique is that when multiple sources produce data on the same sample set, a fusion technique could help in the improvement of further predictor models as well as provide an enhanced interpretation of resulting models. Fusion techniques are particularly important when the different blocks of data have different dimensions. In the latter case, fusion techniques can give equal weight to all data blocks in the new fused dataset. Furthermore, different levels of data fusion can be used: low-level data fusion, mid-level data fusion and high-level data fusion. Low-level fusion refers to combining individual data blocks and then subjecting them to chemometric treatment. Mid-level fusion occurs when unsupervised techniques such as PCA are utilized to extract features that are then concatenated into one data block, where further chemometric treatment can be applied. In high-level fusion, supervised techniques are used on each block separately before combining them into one dataset [35,36].

Data fusion of DAD and MS datasets was carried out using mid-level and high-level data fusion, and PLS-DA was applied as a modeling technique. The results were then compared with the individual DAD and MS models.

### 4.9. Software

The Progenesis QI software from Waters (Milford, MA, USA) was used for data handling of raw MS data. Matlab version 2020b from Mathworks (Natick, MA, USA) was used for data processing and modeling. The ChemoAc toolbox, version 4 (Brussels, Belgium), was used for the application of PLS-DA along with the PLS toolbox version 8.9.2.

## 5. Conclusions

This paper has explored the possibility of combining chromatographic fingerprinting using MS with chemometrics in the domain of plant food supplements constituted of plant mixtures. The approach followed to detect regulated plants in plant food supplements was found to be feasible in practicality, but results have to be confirmed with other, preferably orthogonal, techniques to provide confirmation of the obtained results. However, the implementation of MS methodology should be carefully evaluated, particularly in terms of the initial investment, ongoing maintenance costs, and overall affordability, especially for smaller laboratories with limited budgets. As mentioned above, models based on DAD detection yielded only slightly worse modeling results for the detection of the targeted plants, and therefore, LC-DAD fingerprinting is a valuable alternative for laboratories that have no access to LC-MS/MS.

Anyway, the results of the samples analyzed in the proof of concept give a first indication that these samples do not always contain what they label on their packaging, and therefore, it can be foreseen that there might be a need for stronger control of the products entering the market and finding their way to the consumer via internet or other dubious channels.

## Figures and Tables

**Table 1:** Final conditions for the LC parameters for the four plants (except the gradient for *Garcinia cambogia*) and MS conditions for *Aristolochia fangchi*, *Ilex paraguariensis*, *Hoodia gordonii* and *Garcinia cambogia*.

LC Parameters			
**Column**	Acquity BEH shield RP18, 1.7 µm, 2.1 mm × 100 mm, 130 Å		
**Flow rate**	0.250 mL/min		
**Column temperature**	35 °C		
**Mobile phase A**	0.1% Formic acid in LC/MS grade water		
**Mobile phase B**	0.1% Formic acid in LC/MS grade Methanol		
**Injection volume**	5 µL		
**Concentration of plant reference**	10 mg/mL		
**Gradient**	Time (min)	%A	%B
	0	90	10
	0.5	90	10
	1.5	50	50
	4.5	50	50
	9	10	90
	10.5	10	90
	12	90	10
	13	90	10
**MS Parameters**			
**Capillary voltage**	3 kV		
**Sampling cone**	40		
**Source temperature**	150 °C		
**Desolvation temperature**	500 °C		
***Gas flows***			
**Cone**	50 L/h		
**Desolvation gas**	800 L/h		
**Nebuliser**	6.5 bar		
**Scan range**	50–2000 m/z		
**Scan time**	0.5 s		

**Table 2:** Difference in gradient for Garcinia cambogia.

**Gradient**	**Time (min)**	**%A**	**%B**
	0	50	50
	0.5	50	50
	5	10	90
	10	10	90
	11	50	50
	12	50	50

**Table 3:** PLS compression with PLS-DA treatment for all the regulated plants.

Slimming Plant	Preprocessing	PLS Factors	Cross-Validation (ccr%) with Misclassified Samples Between ()	Modeling (ccr%)	External Test Set Validation (ccr%) with Misclassified Samples Between ()
*Aristolochia fangchi*	1st derivative	3	100% (0/50)	100%	100% (0/17)
*Ilex paraguariensis*	-	2	100% (0/50)	100%	94% (1/17)
*Hoodia gordonii*	1st derivative	2	100% (0/50)	100%	100% (0/17)
*Garcinia cambogia*	-	2	98% (1/50)	94%	94% (1/17)

**Table 4:** Confusion matrix for the four plants.

Slimming Plant	True Positives Training Set Test Set		False Positives Training Set Test Set		True Negatives Training Set Test Set		False Negatives Training Set Test Set	
	(cv)		(cv)		(cv)		(cv)	
*Aristolochia fangchi*	43	13	0	0	7	4	0	0
*Ilex paraguariensis*	41	14	0	0	9	2	0	1
*Hoodia gordonii*	42	14	0	0	8	3	0	0
*Garcinia cambogia*	40	14	1	0	8	2	0	1

**Table 5:** Comparison between DAD models, MS models and fused DAD-MS models with double PLS-DA treatment for each of the targeted plants.

Slimming Plant	Modeling	Preprocessing	PLS Factors	Cross-Validation (ccr%)	Modeling (ccr%)	External Test Set Validation
*Ilex paraguariensis*	DAD-MS Fused model	-	2	92%	94%	88%
	DAD model	-	14	96%	100%	94%
	MS model	-	2	100%	100%	94%
*Hoodia gordonii*	DAD-MS Fused model	1st derivative	2	100%	94%	100%
	DAD model		20	88%	96%	94%
	MS model	1st derivative	2	100%	100%	100%
*Aristolochia fangchi*	DAD-MS Fused model	-	2	92%	92%	100%
	DAD model	-	23	94%	96%	94%
	MS model	1st derivative	3	100%	100%	100%
*Garcinia cambogia*	Fused model	-	2	98%	100%	94%
	DAD model	-	22	96%	98%	88%
	MS model	-	2	98%	94%	94%

**Table 6:** Proof of concept for 10 samples using the MS modeling approach.

Illegal Samples	Sample with Claimed Presence of the Respective Plants	Samples Predicted for *Ilex paraguariensis*	Samples Predicted for *Aristolochia fangchi*	Samples Predicted for *Hoodia gordonii*	Samples Predicted for *Garcinia cambogia*
1	*Garcinia cambogia*	Present	-	Present	-
2	*-*	Present	-	-	-
3	*Ilex paraguariensis*	Present	-	-	-
4	*Ilex paraguariensis*	Present	-	-	-
5	*Garcinia cambogia*	-	-	-	-
6	*-*	-	-	-	-
7	*Garcinia cambogia*	-	-	-	-
8	*Garcinia cambogia*	Present	Present	-	-
9	*-*	-	-	-	-
10	*Garcinia cambogia*	-	-	-	-

**Table 7:** Dimensions of the 3D matrices for all studied plants.

Plant	Data Cube Dimensions (Timepoints × Intensity and Mass Data × Samples)	Unfolded Data
*Aritolochia fangchi*	10,680 × 2 × 67	67 × 21,360
*Ilex paraguariensis*	17,061 × 2 × 67	67 × 34,122
*Hoodia gordonii*	7124 × 2 × 67	67 × 14,248
*Garcinia cambogia*	15,625 × 2 × 67	67 × 31,250

## Data Availability

Data is contained within the article or Supplementary Materials.

## Supplementary Materials

The following supporting information can be downloaded at: https://www.mdpi.com/article/10.3390/molecules30143001/s1, Title 1: Variable importance in projection scores for the four plants along with the comparison with respective MS chromatograms, Figure S1. VIP scores along with MS chromatogram for *Aristolochia fangchi*, Figure S2. VIP scores along with MS chromatogram for *Ilex paraguariensis*, Figure S3. VIP scores along with MS chromatogram for *Hoodia gordonii*, Figure S4. VIP scores along with MS chromatogram for *Garcinia cambogia*. Title 2: Evaluation of PCA and PLS compression by comparing the modeling results, Table S1. Comparison between PCA and PLS compression using modeling for *Ilex paraguariensis*, Table S2. Comparison between PCA and PLS compression using modeling for *Aristolochia fangchi*, Table S3. Comparison between PCA and PLS compression using modeling for *Hoodia gordonii*, Table S4. Comparison between PCA and PLS compression using modeling for *Garcinia cambogia.*

## Abbreviations

The following abbreviations are used in this manuscript:

MS	Mass spectrometry
DAD	Diode array detection
PLS-DA	Partial least squares-discriminant analysis
PCA	Principal component analysis
WHO	World health organization
UHPLC	Ultra-high performance liquid chromatography
LC	Liquid chromatography
UV	Ultra-violet
COW	Correlation optimized warping
RASFF	Rapid alert system of food and feed
Q-tof	Quadrupole time of flight
CCR%	Correct classification rate%
FAMPH	Federal agency for medicines and health products
FASFC	Federal agency for the safety of food chain
VIP	Variable importance in projection
