# Supplementary material for: Mass Spectrometric Fingerprinting to Detect Fraud and Herbal Adulteration in Plant Food Supplements
Source: Molecules. 2025 Jul 17;30(14):3001. doi: 10.3390/molecules30143001 (PMC12298695; doi:10.3390/molecules30143001)
Supplement: Supplementary file 1 [file molecules-30-03001-s001.zip › molecules-3713464-supplementary.pdf]

Supplementary Material

Title 1: Variable importance in Projection scores for the four plants along with the comparison with respective MS chromatograms

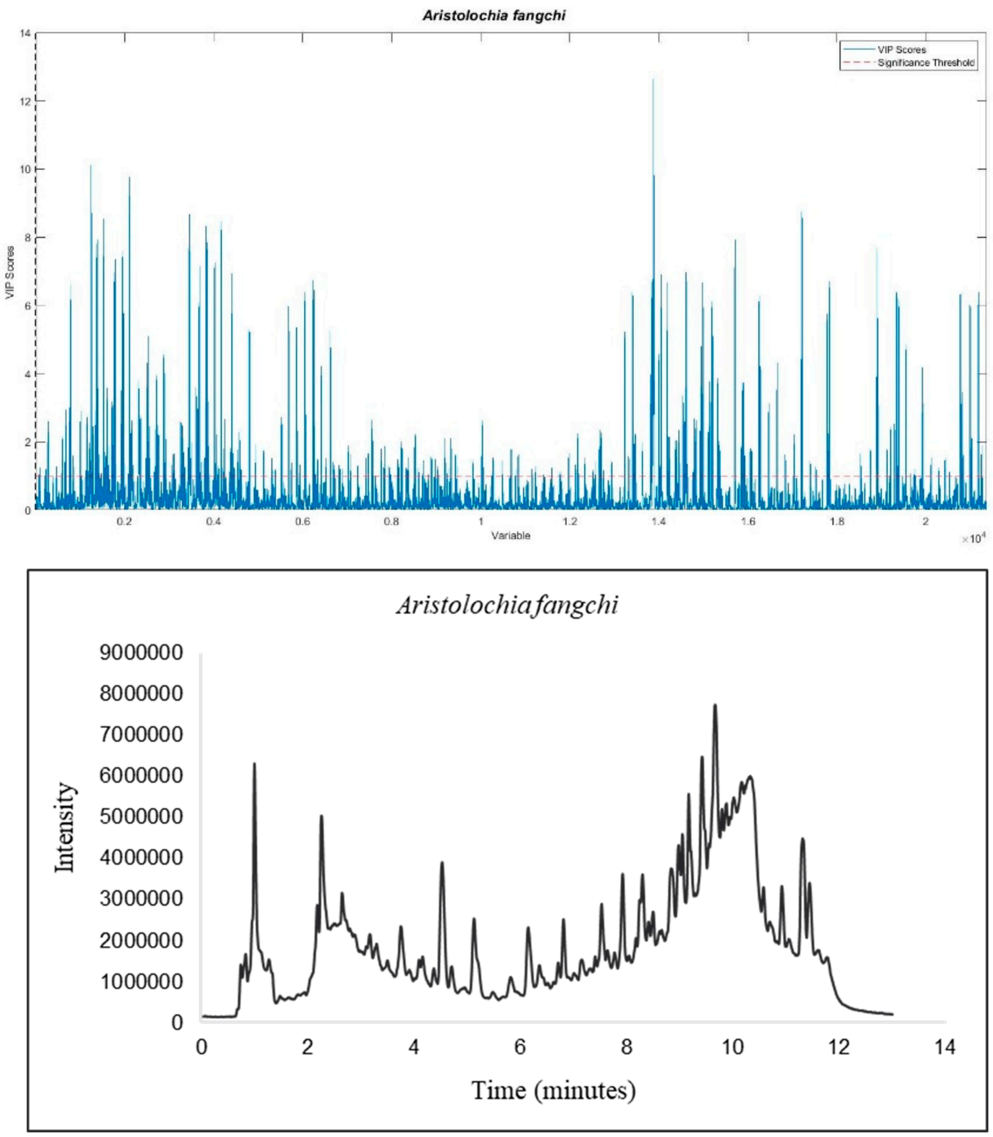

Figure S1 VIP scores along with MS chromatogram for *Aristolochia fangchi*

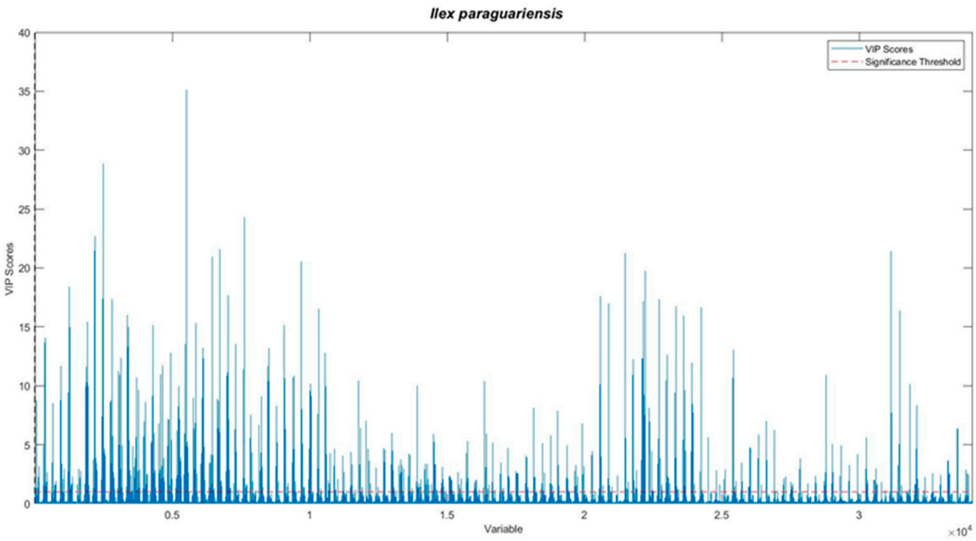

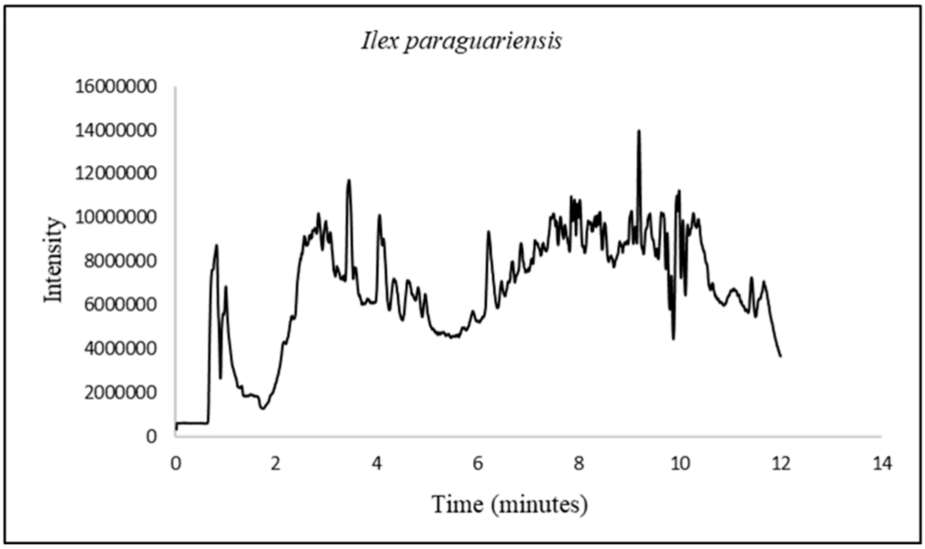

Figure S2. VIP scores along with MS chromatogram for *Ilex paraguariensis*

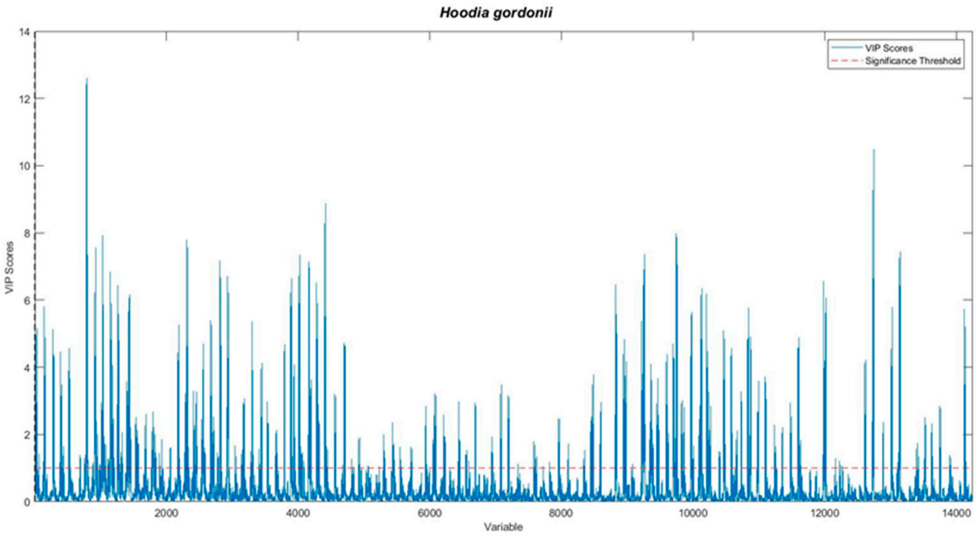

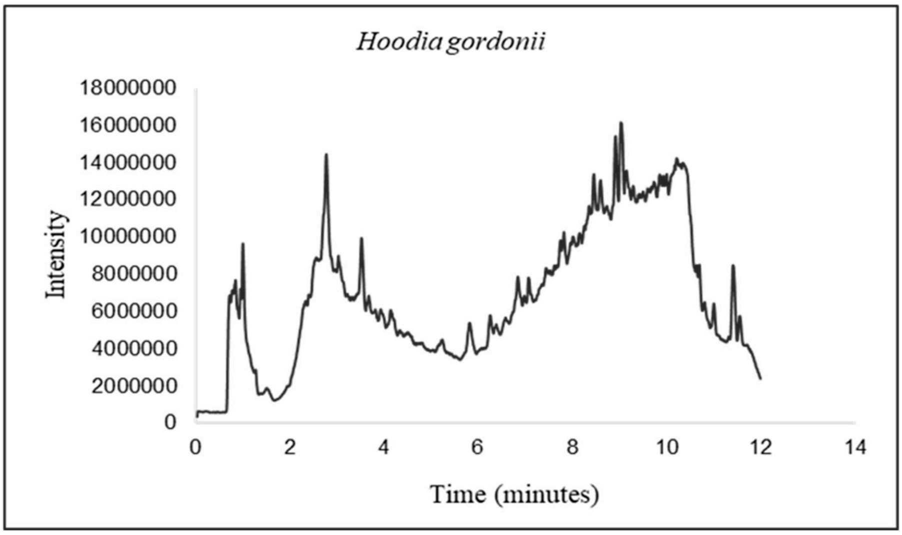

Figure S3. VIP scores along with MS chromatogram for *Hoodia gordonii*

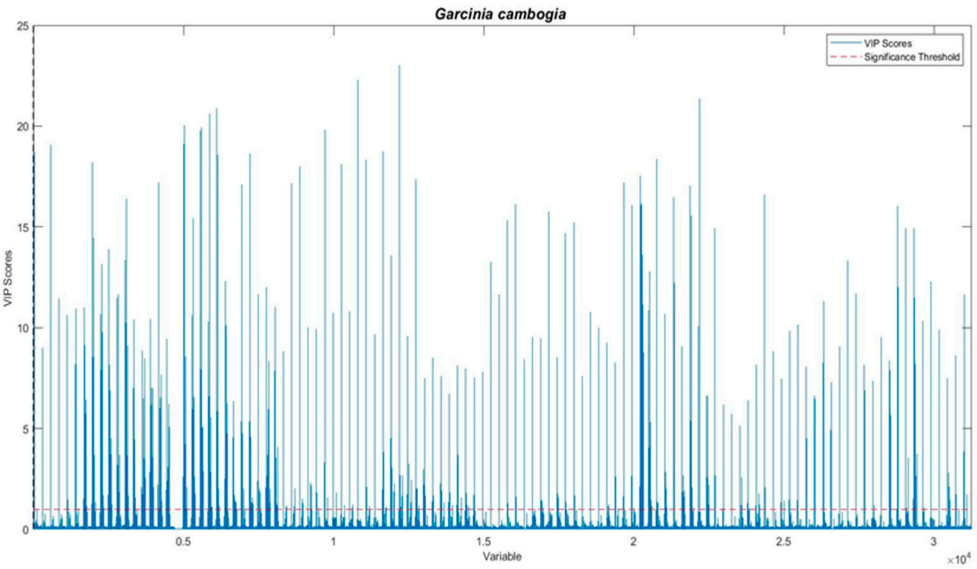

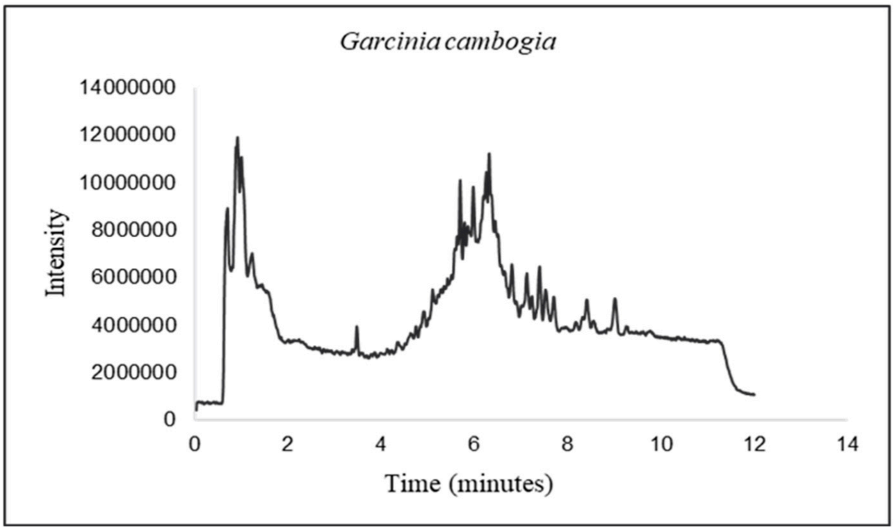

Figure S4. VIP scores along with MS chromatogram for *Garcinia cambogia*

Title 2: Evaluation of PCA and PLS compression by comparing the modelling results

Table S1. Comparison between PCA and PLS compression using modelling for *Ilex paraguariensis*

PLS compression - PLS DA modelling (ccr%)	
**PLS factors**	2
**PLS CV**	100%
**PLS Model**	100%
**External testset prediction**	94%
PCA compression - PLS-DA modelling (ccr%)	
**PLS factors**	2
**PLS CV**	82%
**PLS Model**	94%
**External testset prediction**	88%

Table S2. Comparison between PCA and PLS compression using modelling for *Aristolochia fangchi*

PLS compression - PLS DA modelling (ccr%)	
**PLS factors**	3
**PLS CV**	100%
**PLS Model**	100%
**External testset prediction**	100%
PCA compression - PLS DA modelling (ccr%)	
**PLS factors**	3
**PLS CV**	86%
**PLS Model**	86%
**External testset prediction**	76%

Table S3. Comparison between PCA and PLS compression using modelling for *Hoodia gordonii*

PLS compression - PLS DA modelling (ccr%)	
**PLS factors**	2
**PLS CV**	100%
**PLS Model**	100%
**External testset prediction**	100%
PCA compression - PLS DA modelling (ccr%)	
**PLS factors**	2
**PLS CV**	82%
**PLS Model**	80%
**External testset prediction**	82%

Table S4. Comparison between PCA and PLS compression using modelling for *Garcinia cambogia*

PLS compression - PLS DA modelling (ccr%)	
**PLS factors**	2
**PLS CV**	98%
**PLS Model**	94%
**External testset prediction**	94%
PCA compression - PLS DA modelling (ccr%)	
**PLS factors**	2
**PLS CV**	82%
**PLS Model**	98%
**External testset prediction**	88%
